# Detection of nucleotide-specific CRISPR/Cas9 modified alleles using multiplex ligation detection

**DOI:** 10.1038/srep32048

**Published:** 2016-08-25

**Authors:** R. KC, A. Srivastava, J. M. Wilkowski, C. E. Richter, J. A. Shavit, D. T. Burke, S. L. Bielas

**Affiliations:** 1 Department of Human Genetics, University of Michigan Medical School, Ann Arbor, Michigan, USA; 2 Department of Pediatrics and Communicable Diseases, Division of Pediatric Hematology/Oncology, University of Michigan Medical School, Ann Arbor, Michigan, USA

## Abstract

CRISPR/Cas9 genome-editing has emerged as a powerful tool to create mutant alleles in model organisms. However, the precision with which these mutations are created has introduced a new set of complications for genotyping and colony management. Traditional gene-targeting approaches in many experimental organisms incorporated exogenous DNA and/or allele specific sequence that allow for genotyping strategies based on binary readout of PCR product amplification and size selection. In contrast, alleles created by non-homologous end-joining (NHEJ) repair of double-stranded DNA breaks generated by Cas9 are much less amenable to such strategies. Here we describe a novel genotyping strategy that is cost effective, sequence specific and allows for accurate and efficient multiplexing of small insertion-deletions and single-nucleotide variants characteristic of CRISPR/Cas9 edited alleles. We show that ligation detection reaction (LDR) can be used to generate products that are sequence specific and uniquely detected by product size and/or fluorescent tags. The method works independently of the model organism and will be useful for colony management as mutant alleles differing by a few nucleotides become more prevalent in experimental animal colonies.

CRISPR/Cas9 genome-editing has emerged as a powerful and simple tool that is making many model organisms more genetically tractable^1^,^2^. The nucleotide-level precision of the technique is accompanied by complications in genotyping and colony management^3^. Genetically modified mice, for an example, represent an important model for understanding gene function in development and disease. Mutant mice conventionally generated by insertional mutagenesis or gene-targeting methods have relied on homologous recombination of exogenous DNA fragments^4^. The integrated exogenous DNA can be exploited to facilitate individual animal genotyping and colony management. More recently, CRISPR/Cas9 gene-targeting technology has been developed to efficiently disrupt genes in zygotes or ES cells for the purpose of creating animal models^5^, including organisms that have traditionally been challenging to manipulate genetically^2^,^4^,^5^. DNA double strand breaks created by Cas9 are repaired by non homologous end joining (NHEJ) when a homologous template is not provided^6^. However, in many cases, the small insertions or deletions (in-dels) created by NHEJ DNA repair do not generate a novel restriction site or change the size sufficiently enough to be easily resolved with traditional genotyping strategies^7^.

Based on the many benefits of this CRISPR/Cas9 gene targeting technology, the number of genome edited animal models is rapidly increasing, necessitating cost effective, rapid and sensitive genotyping strategies. Conventional polymerase chain reaction (PCR) followed by agarose gel electrophoresis lacks the sensitivity to detect products that differ by only a few nucleotides. Assays that have been used to detect the small genetic variants created at targeted loci include: Surveyor Assay (Cel1)^8^,^9^,^10^, T7 Endonuclease I^8^,^11^,^12^, high resolution melting curve analysis (HRMA)^13^, allele specific PCR (ASPCR)^14^, restriction fragment length polymorphism (RFLP)^15^ and direct Sanger sequencing of PCR products. While Surveyor and T7 Endonuclease I specifically cleave heteroduplex DNA mismatch, HRMA utilizes the difference in melting curve of the heteroduplex, and RFLP is dependent upon creation or elimination of a defined restriction site within the targeted locus. These assays are expensive, time consuming, lack sequence specificity, and require costly capital equipment. Moreover, several of these techniques fail to distinguish biallelic combinations for all modes of inheritance; wild type, heterozygous, compound heterozygous and homozygous mutations^15^,^16^.

Here, we describe a cost-effective approach to accurately, reliably, and efficiently genotype CRISPR/Cas9 alleles generated in model organisms that only differ by small in-dels. We adapted a PCR ligation detection reaction (PCR-LDR) as a sequence-specific genotyping method that can be completed quickly with a small number of steps^17^,^18^. With this assay, sequence variance is detected when perfectly matched base-pair probes are ligated at the nick junction of two synthetic oligonucleotides using high-fidelity DNA ligase (Fig. 1a). Allele-specific ligation products can be uniquely distinguished by using primers of different lengths and/or fluorescent tag modifications. LDR is straightforward to implement in most molecular biology laboratories, and has the potential to replace extant protocols for routine laboratory genotyping of model organisms.

## Results

### PCR-LDR for genotyping

**Figure 1:**
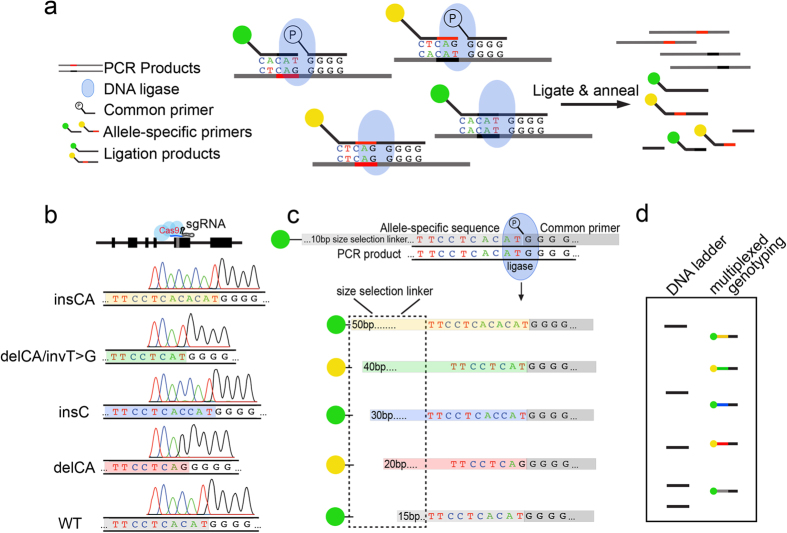
LDR genotyping strategy to detect small in-dels in Asxl3. (**a**) LDR utilizes DNA ligase to ligate together perfectly adjacent primers. Allele-specific primers are designed to complement a common primer that recognizes all alleles similarly. Ligation products are created only when the allele-specific and common primers completely anneal to PCR products amplified from organism to be genotyped. (**b**) *Asxl3* mouse alleles created with CRISPR/Cas9 genome editing. Cas9 nuclease (light sky blue) was targeted to *Asxl3* Exon 12 by a single guide RNA (sgRNA). Target specific double strand DNA cleavage was repaired by non-homologous end joining creating in-dels [CA deletion and T > G transversion (del1), C insertion (ins1), CA deletion (del2), and CA insertion (ins2)] defined by Sanger sequencing. (**c**) Allele-specific and a common primer pair are designed to distinguish specific alleles within each PCR amplicon. The allele-specific and common primers are ligated on complementary amplicons by thermostable ligase (skyblue). Each allele-specific primer is designed with a 5′ size-selection linker of varying nucleotides to modulate the lengths of ligation products and fluorescently labeled FAM- (yellow circle) or TET-tag (green circle). The common primer is phosphorylated at the 5′ end. (**d**) Products of LDR reaction can be separated on an acrylamide gel and uniquely detected based on size and fluorophore.

Mouse lines with frameshift truncating mutations in *Asxl3* were generated using CRISPR/Cas9 genome editing technology. sgRNA targeted to exon 12 of *Asxl3* directed double strand breaks that underwent NHEJ DNA repair creating a series of small in-dels (Fig. 1b). Four founder lines were established that include deletion 1 (del1; a two-nucleotide CA deletion with T > G transversion), deletion 2 (del2; a two-nucleotide CA deletion), insertion 1 (ins1; a single nucleotide C insertion) and insertion 2 (ins2; a two-nucleotide CA insertion) (Supplementary Fig. S1). All four alleles are transmitted in independent mouse lines.

Locus specific PCR amplification generates an amplicon that incorporates the edited region of *Asxl3* exon 12 (Fig. 1c). PCR primers are designed to co-amplify wild type and mutant products from genomic DNA, with the edited regions in the middle of the amplicon. Amplicons are prepared for LDR after protease treatment to inactivate PCR enzymes. LDR achieves linear target product amplification by ligating a pair of target-complementary probes (Fig. 1a). Each probe pair consists of one common universal and one allele-specific probe. The common probe sequence is shared by all *Asxl3* alleles and designed with a 5′ phosphorylation to enable successful ligation. The allele-specific oligonucleotide probes hybridize distinct *Asxl3* mutations and can be uniquely modified by sequence tags that vary in nucleotide length and fluorescent moieties. Our size selection tag sequence was chosen based on lack of complementarity to genomic sequence in the mouse genome. The modifications allow ligation products corresponding to specific alleles to be distinguished by both size and/or fluorescence following acrylamide gel electrophoresis. Unique product sizes and fluorophores also permit multiplexing for simultaneous detection of all expected alleles in a single reaction (Fig. 1d). Size-selection sequence tags on the *Asxl3* allele-specific oligonucleotide probes vary by ~10 nucleotides and fluorescent moieties on the 5′ end alternated between FAM or TET (Supplementary Table 1). If there is target-complementarity between the template PCR strand and probes, ligation of the common and allele-specific probes will be catalyzed by the thermo-stable DNA ligase after repeated cycles of denaturation and ligation (Fig. 1a).

### LDR reaction for genotyping *Asxl3* mutant alleles

**Figure 2:**
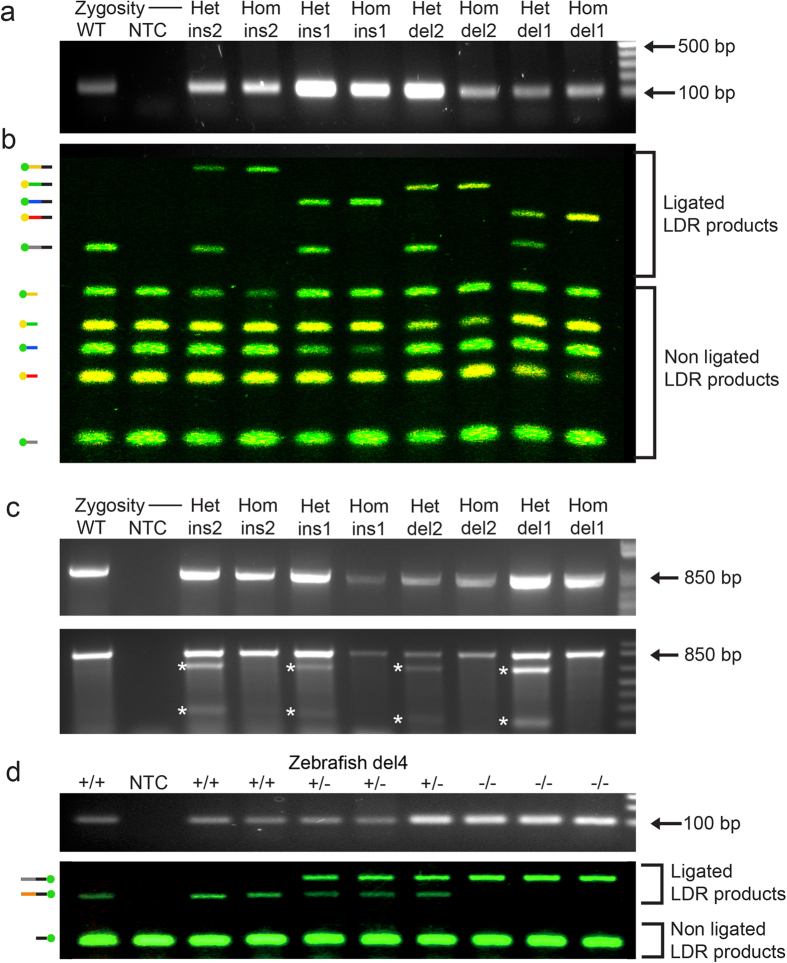
Detection of CRISPR/Cas9 mediated *Asxl3* alleles by LDR genotyping. (**a**) A 110 bp amplicon was PCR amplified from *Asxl3* modified locus. Small *Asxl3* insertions (I) and deletion (D) alleles are not resolved for heterozygous (het) or homozygous (hom) samples following electrophoresis on a 1.5% agarose gel. Empty lane represents no-template control (NTC). (**b**) *Asxl3* allele specific LDR reaction products are uniquely assigned by size and fluorophore following polyacrylamide gel electrophoresis. Bands at the top of the gene represent allele specific ligation products (colored allele specific primer with black common primer) that resolve wild type (WT), heterozygous, and homozygous samples. Shared common bands at bottom of the gel represent unligated primers (colored primers) present in the LDR master mix. The relative fluorophore and size variation of ligated and unligated products are graphically depicted on the left side of the gel. (**c**) A 850 bp amplicon was PCR amplified from *Asxl3* modified locus. *Asxl3* alleles carrying small in-dels are not resolved relative to the WT allele in either heterozygous or homozygous samples following electrophoresis on a 1.5% agarose gel. Renatured PCR products were incubated with T7 Endonuclease I to detect heteroduplex formation. This assay does not differentiate between WT and homozygous *Asxl3* samples as both form homoduplexes not sensitive to T7 Endonuclease I cleavage. Cleavage products (asterisks) are evident in heterozygous samples that form heteroduplexes between mismatched *Asxl3* alleles. (**d**) A 104 bp amplicon was PCR amplified and products checked on 1.5% agarose gel. Ligation product for wt (Z-wt), heterozygous (Z-het) and homozygous 4 bp deletions (Z-del) in a unique sequence in the zebrafish genome were generated by PCR-LDR and uniquely identified by size following PAGE. Ligated LDR reaction products are uniquely identified by size at the top of the gel. The band at bottom of the gel is the unligated 6FAM labeled common primer in the LDR master mix.

To genotype *Asxl3* mutant mice, a 110 bp PCR product was amplified across the site of CRISPR/Cas9 genome editing and separated on a 1.5% agarose gel. Despite the small size of the PCR product, small in-dels <5 nucleotides could not be distinguished from the wild type based on mobility (Fig. 2a). Analysis of the PCR products by polyacrylamide gel electrophoresis (PAGE) was also not sufficient to uniquely identify the different alleles for genotyping purposes (Supplementary Fig. S2). Protease treated PCR products were incubated with a ligase master mix that includes a common probe and allele-specific probes that recognize in-dels del1, del2, ins1 and ins2. Ligation products were analyzed by electrophoresis on a 10% polyacrylamide gel. For each *Asxl3* mouse line, the wild type, heterozygous and homozygous mutant ligation products for each allele were easily distinguished by PAGE (Fig. 2b). Allele-specificity is demonstrated by the different nucleotide sizes of ligation products generated for each allele. The sequence specificity of LDR is indicated by the lack of off-target ligation products from the ligation master mix containing all of the allele-specific probes. Analysis of ligation products indicated that different alleles are easily distinguishable, demonstrating specificity with no evidence of crossover or false positive ligation between ligation primers for different in-del variants. The genotyping strategy clearly differentiates between alleles that differ by a single nucleotide substitution, as in the comparison of alleles del1 and del2 (Fig. 2b, lanes 1–4).

**Figure 3:**
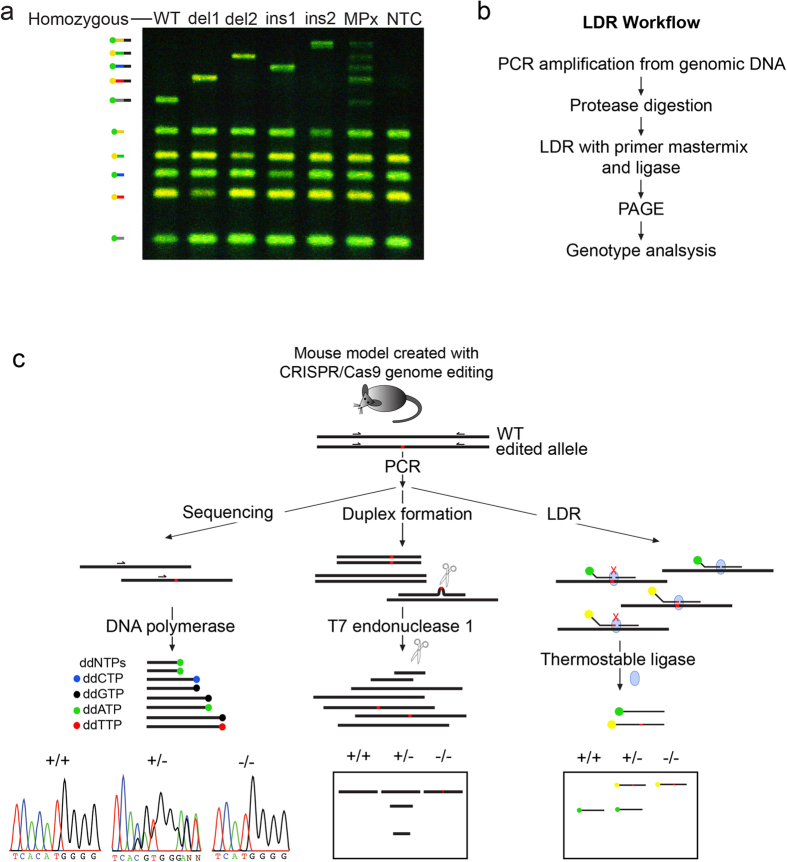
Multi-allelic discrimination using LDR genotyping. (**a**) The specificity of LDR in single and multiplex reactions. *Asxl3* alleles PCR amplified alone or in multiplex can be uniquely detected by LDR. *Asxl3* mouse DNA samples for WT and the four unique homozygous mutant alleles were amplified separately and in combination. LDR products corresponding to the WT and mutant alleles (del1, del2, ins1, ins2) are distinguished by primers ligated on multiplexed (MPx) PCR products. Lanes 1–5 show LDR reaction for individual homozygous alleles, alongside multiplex LDR reaction of all five amplicons together in a reaction. The last lane represents control with no template DNA present (NTC). Bands corresponding to ligation products and unligated allele specific primers are graphically depicted. (**b**) Outline of LDR workflow. (**c**) A schematic comparison of allele detection for genotyping strategies to detect mutations created by CRISPR/Cas9 genome editing at a specific locus. Sanger sequencing clearly defines mutations in homozygous samples. Some ambiguity exists in heterozygous samples. T7 endonuclease can cleave heteroduplexes formed between PCR products from heterozygous mouse samples, but does not distinguish between homozygous WT and mutant genotypes. LDR can uniquely detect small DNA sequence specific differences in all modes of inheritance.

In contrast to the PCR-LDR method, T7 Endonuclease I cleavage techniques fail to resolve WT or mutant homozygous alleles. An ~850 base pair *Asxl3* exon12 PCR product was amplified across the site of genome editing (Fig. 2c). Denatured amplicons were allowed to reanneal with heteroduplexes forming between products with mismatched nucleotides (Fig. 3b). T7 Endonuclease I cleaved PCR products at sites of heteroduplex mismatch, and the resulting smaller fragments were resolved on an agarose gel. T7 Endonuclease I digestion efficiently detects heterozygous animals in all four unique *Asxl3* lines, with smaller cleaved DNA fragments observed in the corresponding lanes. T7 Endonuclease I does not cleave PCR products in homoduplexes and only full length PCR products were detected in homozygous animals. Consequently, the technique fails to distinguish WT or mutant homozygotes (Fig. 2c). The T7 Endonuclease I genotyping strategy is also unable to distinguish between the distinct *Asxl3* alleles, even in heterozygous animals, since the size of the cleavage products are the same for the different *Asxl3* alleles.

### Zebrafish LDR-PCR genotying

To test the general application of our technique between species, this genotyping strategy was used for a zebrafish line that was CRISPR/Cas9 genome-edited at a unique sequence in the zebrafish genome. The PCR-LDR genotyping strategy was established to detect a four base pair deletion (Z-del) segregating in the colony (Supplementary Fig. S3a). For the LDR reaction, common primers were designed with the 6FAM florescent moiety and allele-specific primers for Z-wt and Z-del alleles were designed with size-selection linkers (Supplementary Table S1). PCR primers were designed to specifically amplify 104 bp fragments flanking the mutated region (Fig. 2d). PCR products resolved on an agarose gel did not distinguish the genotypes of the samples by size. PCR products of Z-het samples could be resolved by size when analyzed by PAGE, yet homozygous Z-wt and Z-del samples were difficult to differentiate by this method (Supplementary Fig. S3b). Zebrafish samples were sequenced and analyzed by PCR-LDR genotyping. Ligation products of LDR and sample genotypes were easily resolved by PAGE (Fig. 2d). Likewise, PCR-LDR and Sanger sequencing identified all genotypes with 100% consistency, but heterozygotes were more clearly genotyped by LDR (Supplementary Fig. S3).

### LDR distinguishes multiplexed alleles

The efficiency of CRISPR/Cas9 genome editing permits simultaneous targeting of both alleles of the same gene. We therefore investigated the potential of LDR genotyping to detect multiple alleles in a single reaction. DNA samples containing *Asxl3* alleles del1, del2, ins1, ins2, WT were combined and the PCR-LDR assay was performed as described. *Asxl3* ligation products were compared with DNA samples from animals homozygous for *Asxl3* for the corresponding alleles. The multiplexed PCR-LDR reaction reliably resolved distinct alleles, as depicted by the ligation products generated after acrylamide gel electrophoresis (Fig. 3a). The signal intensity of each band was sufficient for detection and all of the bands were of similar signal intensity, suggesting that all ligation products are equivalently generated. Because each allele can be resolved by simple visual examination, this strategy can also be employed for genotyping compound heterozygotes when different *Asxl3* lines are intercrossed.

## Discussion

PCR-LDR is an inexpensive and timely genotyping strategy capable of detecting small genetic changes in multiple alleles (Table 1). PCR-LDR uniquely detects individual alleles so that compound heterozygotes can be genotyped in a single reaction, which is difficult with T7 Endonuclease I or Sanger sequencing (Fig. 3b). The PCR-LDR genotyping strategy overcomes the deficiency in distinguishing between different homozygous genotypes encountered with T7 Endonuclease I assays. Because T7 Endonuclease I indiscriminately cleaves heteroduplexed PCR products with nucleotide mismatches, genotyping based on T7 endonuclease I must be periodically confirmed by sequencing, adding cost and time to experiments and colony management. While sequencing is unparalleled in specificity to define sequence differences between alleles, it is the most expensive and time-intensive genotyping option (Table 1). In addition, sequencing of heterozygous alleles can be difficult to interpret due to overlapping fluorochrome peaks in chromatographs, a problem amplified for compound heterozygote genotypes. PCR-LDR is an inexpensive genotyping strategy that can differentiate between multiple alleles that differ only by small in-dels or single nucleotide polymorphisms. The LDR strategy is valuable for routine genotyping, however, it should be noted that it cannot be used for screening initial mutations as it requires prior sequence information. While the utility of the LDR genotyping strategy has been demonstrated for CRISPR/Cas9 mutated genomes, this strategy should be broadly applicable for other small mutations created by genome editing techniques such as TALENs and ZFNs. The LDR approach for genotyping small variations created by different nucleases has the potential to become an essential laboratory tool for regularly maintaining alleles and managing colonies.

## Materials and Methods

### Animal Models

Mouse colonies were maintained in standard cages in the University of Michigan animal facility on a daily 12 hours light dark cycle. Zebrafish were raised at 28.5 °C with a 14-hour light/10-hour dark cycle. All experiments were performed in accordance with animal protocols approved by the Unit for Laboratory Animal Medicine (ULAM), University of Michigan.

### Genomic DNA extraction

Genomic DNA was isolated from mouse-tail biopsies using simple lysis-extraction procedures. Each tail clipping was lysed in 100 μl of lysis buffer (NaOH, 25 mM, Na_2_EDTA.2H_2_O, 0.2 mM) at 95 °C for 30 mins–1 hr, and neutralized with an equal volume of Tris-Cl (40 mM), and briefly centrifuged. 1 μl of final supernatant was used for each PCR reaction.

### T7E1 Assay

PCR primers (Supplementary Table S1) were designed to amplify a product size of ~850 bp flanking the targeted mutation site. T7 Endonuclease I assay was performed on the PCR products according to the manufacturer’s instruction (Ipswich, MA, USA). The reaction products were resolved using 1.5% agarose gel electrophoresis.

### PCR and Probe Primer Design

PCR primers (Supp. Table1) were designed to amplify a region of *Asxl3* exon 12 with the product size of ~110 bp. The genotyping specificity of the LDR assay is attained by ensuring the stringency of primer design and reaction conditions. LDR probes were designed to distinguish specific alleles within each PCR amplicon by the identity of the fine nucleotides in the probe. The sequence of each allele-specific primer differs for each allele and is labeled at the 5′ end with a 6FAM or TET fluorescent moiety. The inclusion of different fluorophore dyes on the specific 5′ primers enables simultaneous multicolor detection of the alleles. The locus-specific common LDR primers were phosphorylated at the 5′ end. Linker nucleotides were appended to the discriminating primers at their 5′ end to modulate the lengths of the expected ligation products. Both PCR and LDR primers were obtained from Integrated DNA Technologies (Coralville, IA).

### PCR amplification and LDR reaction

The PCR reaction to amplify 110 bp fragment and 850 bp fragment was carried out in 20 μl reaction with primers listed in supplementary table S1, using 10 μl of GoTaq Green Master Mix (M7123, Promega, Madison WI) 0.4 μM each of forward and reverse primers and 1 μl of genomic DNA template. Thermocycling conditions were: 94 °C for 4 min; 94 °C for 30 sec, 60 °C (pre-LDR) and 56 °C (pre-T7E1) for 30 sec; 72 °C for 30 sec for 20–35 cycles; and a 5 min final extension at 72 °C. The 15 μl PCR products were treated with 50 μg protease (19155, Qiagen, Germantown, MD) for 20 min at 50 °C and inactivated at 75 °C for 15 min (Supplementary Fig. S4). The LDR was carried out in 20 μl reaction consisting of 10 μl of protease treated PCR product and 10 μl of LDR master mix (0.25 uM of each LDR primers, 20 mM Tris (20 mM MgCl2, 40 mM KCl, 2 mM NAD) pH 8.3, 0.01% of TritonX-100 and 0.1 μl thermostable ligase (Amphiligase (A8101, Epicentre, Madison, WI; Oakland Genetics, Rochester Hills, MI, www.oaklandgenetics.com) or 0.1 μl ligase expressed and isolated in-house). LDR Thermal cycling parameters: 94 °C for 3 min, 20–30 cycles of 94 °C for 15 sec and 61 °C for 1 min. PAGE was performed at 250 volts using a 15 cm long 10% polyacrylamide non-denaturing gel (12.5 mL 40% Acrylamide/Bis-solution 19:1 (Biorad 1610144), 500 μl of fresh 10% APS and 50 μl of TEMED and 1XTBE to 50 mL) in 1XTBE for 50 min, with compatible gel electrophoresis apparatus (Biometra V15.17, Germany).

### PAGE analysis of PCR products

PCR products were directly loaded into 15% non denaturing polyacrylamide gel Acrylamide/Bis-solution 19:1 (Biorad 1610144) along with the DNA ladder (ThermoFisher 10787018). 1.5 m spacer used for casting the gel with 1X Tris-borate-EDTA (TBE), ammonium persulfate, and TEMED. After 2 hours of electrophoresis in 1X TBE at 150 V the gel was incubated for 2 mins at room temp in 1:10,000 ethidium bromide solution and washed once with 1X TBE for visualization.

## Additional Information

**How to cite this article**: KC, R. *et al*. Detection of nucleotide-specific CRISPR/Cas9 modified alleles using multiplex ligation detection. *Sci. Rep.* **6**, 32048; doi: 10.1038/srep32048 (2016).

## Supplementary Material

Supplementary Information

## Figures and Tables

**Table 1:** Genotyping methods compared.

Method			LDR	Endonuclease Digestion (T7E1/Surveyor)	Sanger Sequencing	Preferred Option
Sample cost ($)			$1	$2–4	$3–5	**LDR**
Total time (hrs)			6–8 hrs	6–12 hrs	24–72 hrs	**LDR/T7E1**
Screening of edited alleles			−	+++	+++	**T7E1/Sanger**
Specificity	Zygosity	hom	+++	−	+++	**LDR/Sanger**
		het	++	++	++	**LDR**
		c-het	+++	+	+	**LDR**

+++High. ++Moderate. +Low. −Not interpretable. $ – Dollars. hrs – hours.
