# Supplementary material for: Detection of nucleotide-specific CRISPR/Cas9 modified alleles using multiplex ligation detection
Source: Sci Rep. 2016 Aug 25;6:32048. doi: 10.1038/srep32048 (PMC4997339; doi:10.1038/srep32048)
Supplement: Supplementary Information [file srep32048-s1.pdf]

Detection of nucleotide-specific CRISPR/Cas9 modified alleles using multiplex ligation-detection

KC R [1], Srivastava A[1], Wilkowski JM [1], Richter CE[2], Shavit JA[2], Burke DT [1], Bielas SL[1]*

[1]Department of Human Genetics, University of Michigan Medical School, Ann Arbor, Michigan, U.S.A.

[2]Department of Pediatrics and Communicable Diseases, Division of Pediatric Hematology/Oncology, University of Michigan Medical School, Ann Arbor, Michigan, U.S.A.

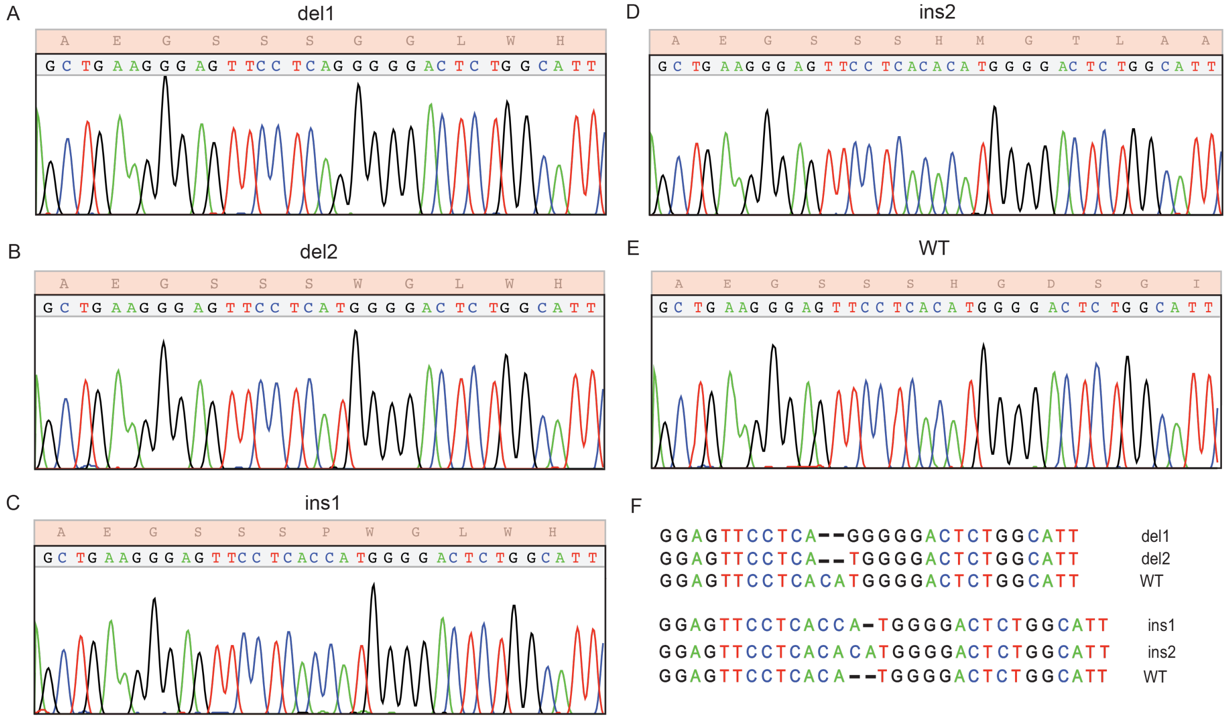

Supplementary figure S1. *Asxl3* mouse alleles created with CRISPR/Cas9 genome editing.

	Oligonucleotide Description	Sequence	Amplicon size
PCR primers	Asxl3 F	ATCGGGCATGTCCAGAGAAG	110
	Asxl3 R	AGCAGAAGGACCAGGAATG	
	Asxl3 T7E1 F	TCACATGGCTTAGTGGTTGT	850
	Asxl3 T7E1R	CTGTTCTTCGGGGTCACTCT	
	Zdel F	TTTCCTCCACAAGTTCTGC	104
	Zdel R	GCTCATTGCTTGATCTTTTCC	
LDR Primers	Asxl3 LDR Wt	/56-FAM/cAGCTGAAGGGAGTTCCTCACAT	59
	Asxl3 LDR del1	/5TET/ctacgtacgtGGAGCTGAAGGGAGTTCCTCAG	68
	Asxl3 LDR ins1	/56-FAM/cgtacgtacgtacgtGGAGCTGAAGGGAGTTCC TCACCAT	76
	Asxl3 LDR del2	/5TET/ctacgtacgtacgtacgtacgtacgtGGAGCTGAAGGGAG TTCCTCAT	84
	Asxl3 LDR ins2	/56-FAM/cgtacgtacgtacgtacgtacgtacgtacgtGGAGCTG AAGGGAGTTCCTCACACAT	93
	Asxl3 LDR common	/5Phos/GGGGACTCTGGCATTCCTGGATGCTCAGACA CAATT	
	Zdel LDR Wt	caagTTCTGCTATATCCACCGTCA	52
	Zdel LDR Mut	tgcttgtttcctccaccaagTTCTGCTATATCCACC	64
	Zdel LDR common	/5Phos/AACATATCCCTTCATCCAACTGAAGAGGT/6FA M/	

Supplementary table S1. Primers and Probe sequences

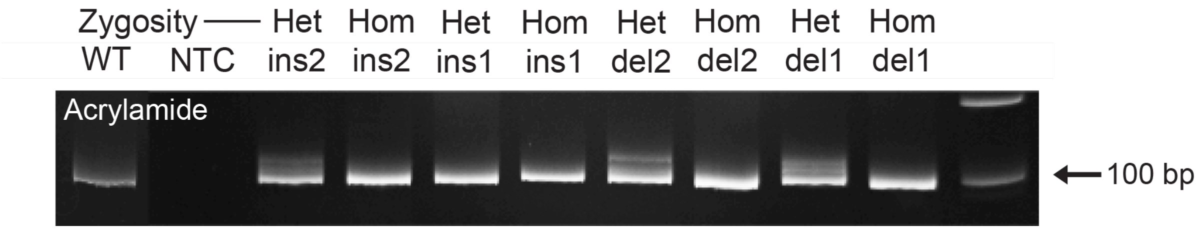

Supplementary figure S2. Separation of 110bp Asxl3 PCR amplified products in 15% non-denaturing acrylamide gel.

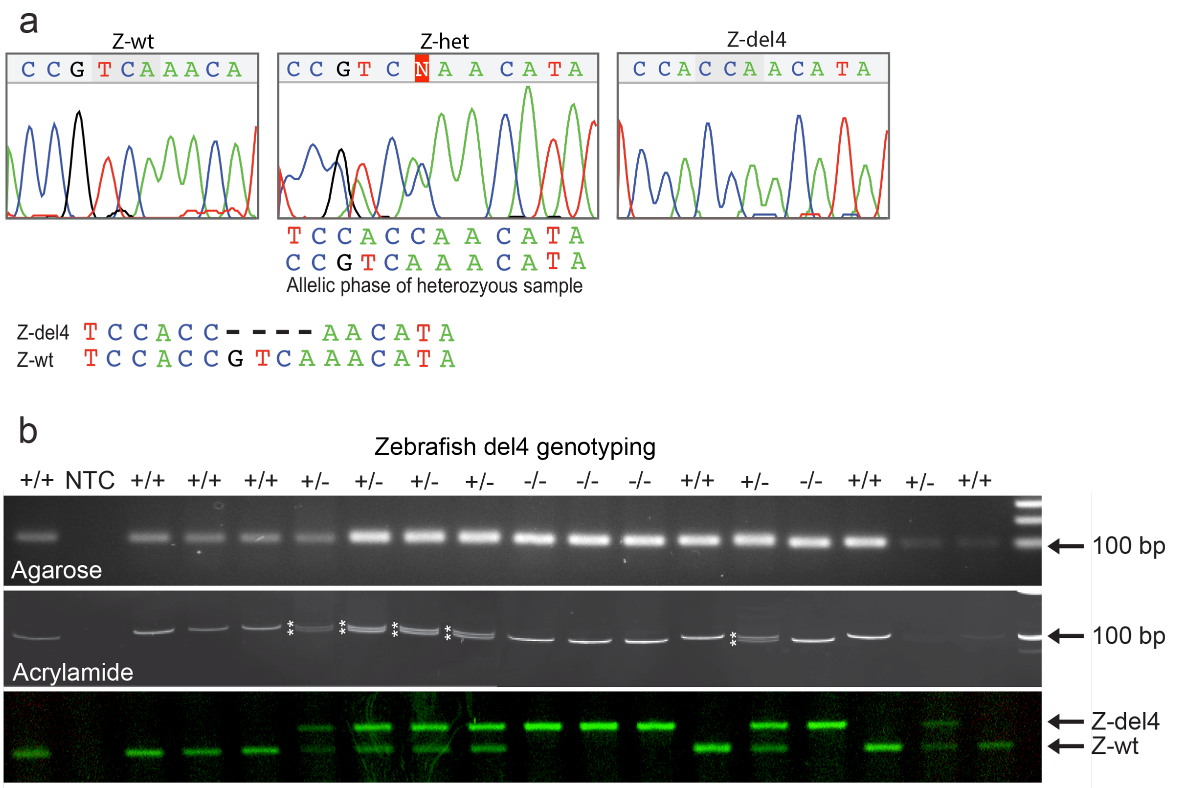

Supplemental figure S3. Detection of CRISPR/Cas9 mediated zebrafish alleles by LDR genotyping. (**a**) Chromatograms showing a 4bp deletion in the zebrafish CRISPR/Cas9 targeted locus. Sequence of Z-del and Z-wt alleles compared defining deleted nucleotides. Predicted allelic phase of Z-het sample depicted below chromatograms based on Z-wt and -del4 homozygous chromatograms. (**b**) Detection of 104 bp amplification products on 1.5% agarose and 15% non-denaturing acrylamide gels. Empty lane represents no template control (NTC). Homozygous Z-wt and Z-del4 PCR products that differ by 4bps are difficult to differentiate between on the acrylamide gel for genotyping purposes. Allele specific LDR reaction products are uniquely assigned by size and detected by fluorophore following polyacrylamide gel electrophoresis, enabling simple and clear genotypic readouts.

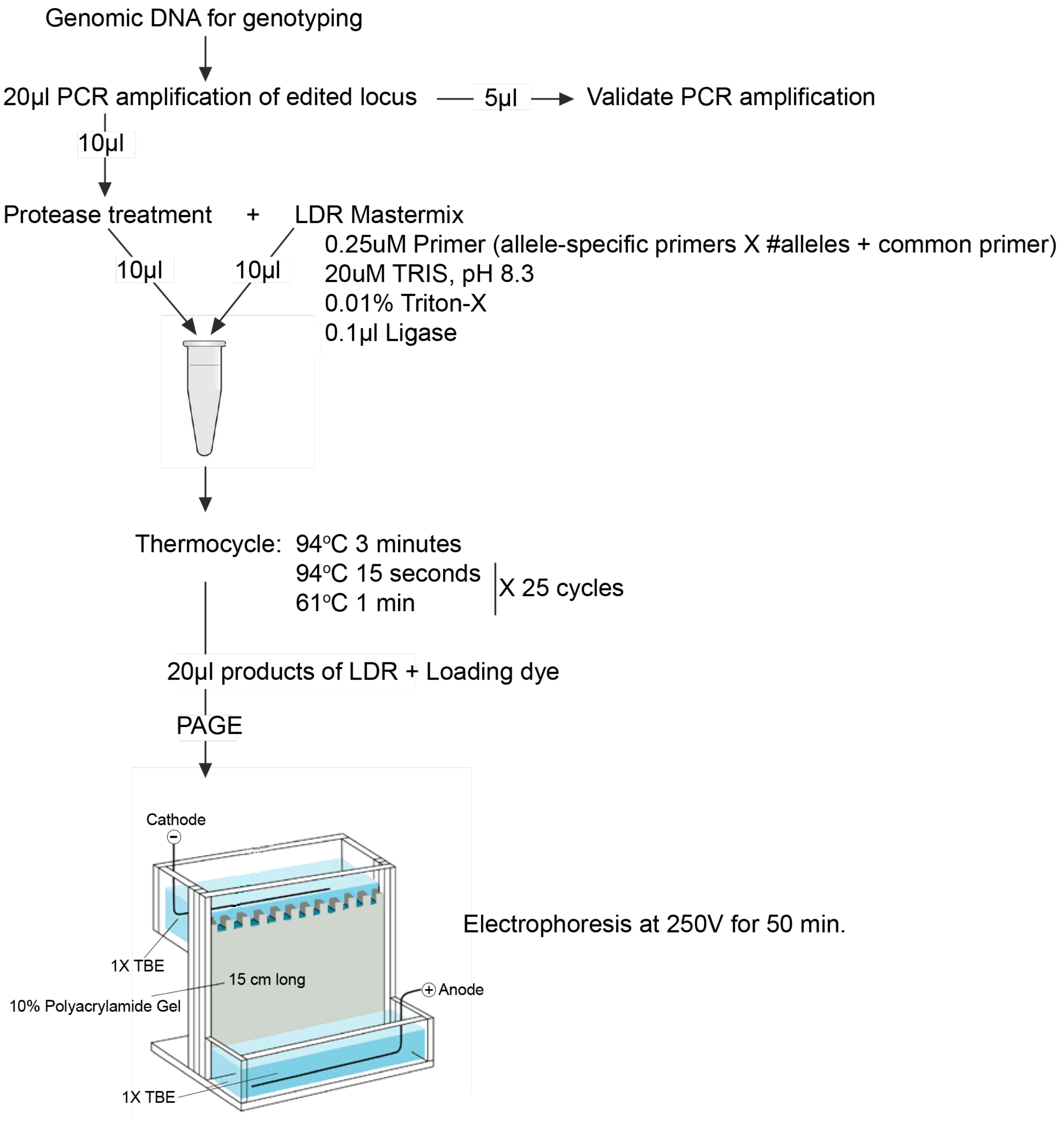

Supplementary figure S4. LDR protocol summary
